# A Defective Circulating Mitochondrial Bioenergetics Profile Reflects the Hepatic One and Outlines Genetic MASLD

**DOI:** 10.3390/antiox14060618

**Published:** 2025-05-22

**Authors:** Erika Paolini, Miriam Longo, Marica Meroni, Paola Podini, Marco Maggioni, Angelo Quattrini, Anna Ludovica Fracanzani, Paola Dongiovanni

**Affiliations:** 1 Medicine and Metabolic Diseases, Fondazione IRCCS Ca’ Granda Ospedale Maggiore Policlinico, 20122 Milan, Italy; erika.paolini@policlinico.mi.it (E.P.); miriam.longo@policlinico.mi.it (M.L.); marica.meroni@policlinico.mi.it (M.M.); anna.fracanzani@unimi.it (A.L.F.); 2 Neuropathology Unit, Institute of Experimental Neurology, IRCCS San Raffaele Scientific Institute, 20132 Milan, Italy; podini.paola@hsr.it (P.P.); quattrini.angelo@hsr.it (A.Q.); 3 Division of Pathology, Fondazione IRCCS Ca’ Granda Ospedale Maggiore Policlinico, 20122 Milan, Italy; marco.maggioni@policlinico.mi.it; 4 Department of Pathophysiology and Transplantation, University of Milan, 20122 Milan, Italy

**Keywords:** MASLD, genetics, mitochondria, non-invasive biomarker

## Abstract

Genetics and mitochondrial (mt) dysfunction contribute to metabolic dysfunction-associated steatotic liver disease (MASLD). Recently, we demonstrated that the co-presence of *PNPLA3*, *TM6SF2* and *MBOAT7* polymorphisms predisposes to disease progression in MASLD patients and that their deletion triggers mt maladaptation in vitro. Here, we deepened the impact of the silencing of these genes on mt dynamism and respiration by reintroducing TM6SF2 and/or MBOAT7 wild-type proteins in deleted cells through lentiviral infection. Since hepatic mt bioenergetics is impaired in MASLD, in the attempt to identify a non-invasive signature, we then compared the enzymatic mt activity of seahorses, which was assessed in liver biopsies and peripheral blood mononuclear cells (PBMCs) of biopsy-proven MASLD patients (n = 44; Discovery cohort) stratified according to the number of the three at-risk variants (3NRV). Concerning the in vitro results, the rescue of MBOAT7 and/or TM6SF2 wild-type proteins resulted in the assembly of spaghetti-shaped mitochondria with improved oxidative phosphorylation (OXPHOS) capacity. In the Discovery cohort, the hepatic bioenergetic profile fully reflected that in PBMCs and was impaired especially in 3NRV carriers. A lowered serum respiration rate was confirmed in noninvasively assessed MASLD (n = 45; Fibroscan-MASLD cohort), while it did not change in unrelated liver disease patients (n = 45). In summary, we firstly demonstrated that mt circulating respirometry reflects that in liver and is specific in defining genetic MASLD.

## 1. Introduction

Over the past two decades, metabolic dysfunction-associated steatotic liver disease (MASLD) depicted a growing burden on health care globally, affecting both children and adults [1,2]. MASLD covers a wide spectrum of chronic liver disorders, ranging from simple steatosis to metabolic dysfunction-associated steatohepatitis (MASH) and fibrosis. In 10–20% of cases, MASH, which is depicted by the presence of inflammation and ballooning, can progress to cirrhosis and hepatocellular carcinoma (HCC), and nowadays, it represents the third leading cause of liver transplantation (LT) [3,4,5]. MASLD is a multifactorial disease whose pathogenesis is shaped by both environmental and genetic factors. Its epidemiology is closely interconnected with epidemic obesity and type 2 diabetes mellitus (T2DM), and it is related to metabolic syndrome (MetS) which includes an umbrella of metabolic conditions such as insulin resistance (IR), dyslipidemia, hyperglycemia, hypertension, and cardiovascular disease (CVD) [6,7].

In the liver mitochondria range of 500 to 4000 number per hepatocyte, these organelles are essential to guarantee whole-body homeostasis and liver bioenergetic metabolism via oxidative phosphorylation (OXPHOS), β-oxidation, the tricarboxylic acid (TCA) cycle and ketogenesis [8,9,10,11]. Mitochondria originate through a self-renewal route defined as mitobiogenesis, and their shaping leads to the fusion–fission interchange regulated by the peroxisome proliferator-activated receptor (PPAR)-γ coactivator 1 (PGC1α) which also manages OXPHOS capacity, heme biosynthesis, glucose and lipid metabolism [12]. During steatosis onset, mitobiogenesis was adapted to counteract fat accumulation by harmonizing fusion, fission and the mitophagyon response to high fat accumulation by harmonizing fusion, fission and mitophagy [13]. However, the increased lipid loading unbalances mitochondrial flexibility, resulting in the assembly of misshapen and unfunctional mitochondria featured by impaired β-oxidation, bioenergetic activity, ketogenesis and adenosine triphosphate (ATP) production. The inhibition of mitophagy at the quality check stage adjuvates the overload of failed organelles, which in turn heightens hepatocellular injury by triggering oxidative stress, inflammation and the release of mitochondrial damage-associated molecular patterns (Mito-DAMPs) encompassing mitochondrial DNA (mtDNA) fragments (ccf-mtDNA) [14]. Moreover, the dysfunctional mitochondria further contribute to reactive oxygen species (ROS) production, including superoxide radicals and hydrogen peroxide (H_2_O_2_), both defined as by-products of the mitochondrial electron transport chain. The exacerbated oxidative stress further compromises the mitochondrial functions, resulting in high hepatocellular damage, lipid peroxidation and inflammation, which in turn trigger MASLD progression [15].

Recently, an oral, liver-directed, thyroid hormone receptor agonist named Resmetirom has been approved by the FDA for the treatment of progressive MASLD. Indeed, data from a Phase 3 trial showed that 29% and 25% of patients who received Resmetirom had a resolution of MASH and an improvement of at least one stage of fibrosis, respectively [16], although follow-up studies are required to confirm its efficacy. Resmetirom represents the first and only drug which has been approved for the treatment of MASH. Lifestyle interventions encompassing a low-calorie diet and physical activity are the still the mainstays of disease treatment, albeit the scant compliance of patients outlines the urgent need for MASLD pharmacotherapy [17]. To date, hepatic biopsy is considered the gold standard diagnostic approach to delineate the degree of steatosis, necroinflammation and ballooning through the scoring system NAFLD Activity Score (NAS) [18,19]. Nonetheless, it exhibits several limitations due to its low applicability, high cost, sample size and repeatability, which in turn decline the accuracy of recognizing first- and end-stage MASLD in some patients, including children. Hence, to face the growing prevalence of MASLD paralleling with the scant diagnostic and therapeutic strategies, newly non-invasive methodological approaches have been proposed to estimate disease severity. Considering mitochondrial maladaptation as a huge hallmark of MASLD progression, emerging evidence highlighted how the release of ccf-mtDNA from hepatocytes into the circulation accurately might estimate organelles’ dysfunction alongside advanced liver injury, thus representing a promising non-invasive tool to identify patients at risk of progressive MASLD [14]. In keeping with this theory, the focus in recent years shifted to explore real-time energy changes in both hepatic and peripheral blood [20,21].

The hereditary component of MASLD has long been established, and 50–70% of individuals susceptibility to develop the disease alongside its phenotypic variability are owed to inherited risk factors [22]. Recently, we have demonstrated that the co-presence of single nucleotide polymorphisms (SNPs) in patatin-like phospholipase domain-containing 3 (*PNPLA3*), transmembrane 6 superfamily member 2 (*TM6SF2*) and membrane bound o-acyltransferase domain-containing 7 (*MBOAT7*) hugely predisposes patients to advanced diseases, highlighting the relevance of polygenic risk scores to identify at-risk individuals [23,24,25]. The impact of these genetic variations alone or combined with MASLD pathogenesis has been investigated in hepatoma cells (HepG2) homozygous for the I148M PNPLA3 variant which were knocked-out (KO) for *MBOAT7* (*MBOAT7^−/−^*), *TM6SF2* (*TM6SF2^−/−^*), or both genes (*MBOAT7^−/−^TM6SF2^−/−^*) through the clustered regularly interspaced short palindromic repeats/CRISPR-associated protein 9 (CRISPR/Cas9) technology in order to mimic the human proteins’ loss-of-function phenotype. Interestingly, we found that the co-silencing of these genes (double KO) alters the mitochondrial morphology and architecture, increases the organelles number and prompts oxidative stress, thus driving MASLD progressive forms [23,26]. Consistently, it has been observed in Huh-7 hepatoma cells that the overexpression of the PNPLA3 I148M variant was correlated with high levels of lactate and γ-glutamyl amino acids, a hallmark of metabolic reprogramming and mitochondrial dysfunction [27]. Concerning the MBOAT7 rs641738 loss-of-function mutation, the enrichment of saturated lipids in membrane affects their composition and dynamics, possibly leading to failed organelle functionality [28,29]. Moreover, the deficiency of TM6SF2 in Hu-7 cells and human liver reflects a reduction in omega-3 polyunsaturated fatty acids (PUFAs) and the accumulation of saturated and monounsaturated ones, thus impairing VLDL harvest and enhancing the assembly of TG and cholesterol–esters compounds. This results in failed mitochondrial β-oxidation, a higher number of lysosomal/endosomal compartments and impaired organelles membrane dynamics [30,31]. In keeping with these data, it has been observed that the lack of TM6SF2 in the small intestine of zebrafish and in Caco-2 enterocytes promotes ER stress by altering lipid clearance and tampers the ER architecture by appearing with enlarged cisternae, supporting the notion that the TM6SF2 loss-of-function mutation may impact the organelles’ morphology [32].

Therefore, to deepen the genetics engagement in mitochondrial dysfunction, in the present study, we firstly evaluated mitochondrial dynamics in term of the fusion/fission balance in HepG2 KO models. Secondly, we overexpressed the wild-type (WT) proteins in KO cells to explore whether TM6SF2 and/or MBOAT7 restoration improves mitochondrial dysfunction in terms of morphology and function. To this purpose, we explored the mitochondrial lifecycle and activity alongside hepatocellular damage and metabolic reprogramming. Finally, from the translational perspective, to use circulating mitochondrial biomarkers for non-invasively predicting disease severity in genetically predisposed individuals, we compared the hepatic and peripheral bioenergetic profiles in MASLD patients carrying *PNPLA3*, *MBOAT7* and/or *TM6SF2* variations.

## 2. Materials and Methods

### 2.1. Discovery Cohort

We evaluated ROS and H_2_O_2_ production; mitochondrial complexes I, III and IV; and citrate synthase kinetic activities as well as respiration capacity in both frozen liver biopsies and peripheral blood mononuclear cells (PBMCs) of 44 MASLD patients who were enrolled consecutively at the Metabolic Liver Diseases outpatient service at Fondazione IRCCS Cà Granda, Ospedale Maggiore Policlinico Milano (Milan, Italy). Inclusion criteria were the availability of a liver biopsy specimen for suspected MASH or severe obesity, DNA samples and clinical data. MASH was defined by the concomitant presence of steatosis, lobular inflammation and hepatocellular ballooning. Individuals with excessive alcohol intake (men, >30 g/d; women, >20 g/d), viral and autoimmune hepatitis or other causes of liver disease were excluded. Informed written consent was obtained from each patient, and the study protocol was approved by the Ethical Committee of the Fondazione and conforms to the ethical guidelines of the 1975 Declaration of Helsinki. The demographic, anthropometric and clinical data of these patients are listed in Table S1. MASLD patients were stratified according to the number of risk variants (NRVs) as follows: 0 for patients who had no risk variants; PNPLA3, MBOAT7 or TM6SF2 for the presence of GG, TT and TT alleles in a homozygous state in the *PNPLA3*, *MBOAT7* and *TM6SF2* genes, respectively (1 NRV); and 3NRV for subjects carrying all 3 at-risk variants in either a heterozygous or homozygous state. PBMCs were extracted from the whole blood of MASLD patients and resuspended in MAS buffer whether liver biopsies were directly homogenate in MAS buffer as described below.

### 2.2. Fibroscan-MASLD Cohort

We assessed the respiration capacity in the frozen PBMCs of 45 patients with a non-invasive diagnosis of MASLD, which was performed by ultrasound echography using a convex 3.5 MHz probe and by FibroScan^®^ (Echosense, Paris, France) (Fibroscan-MASLD) at the Metabolic Liver Diseases outpatient service at Fondazione IRCCS Cà Granda, Ospedale Maggiore Policlinico Milano (Milan, Italy). Informed written consent was obtained from each patient, and the study protocol was approved by the Ethical Committee of the Fondazione IRCCS Ca’ Granda, Milan, and conforms to the ethical guidelines of the 1975 Declaration of Helsinki. The demographic, anthropometric and clinical data of these patients are listed in Table S2. The clinical features of the Fibroscan-MASLD cohort were matched for age, sex and BMI with patients of the Discovery cohort. Patients were stratified according to the number of risk variants (NRVs), as described for the Discovery cohort.

### 2.3. Unrelated Liver Disease Cohort

We evaluated the respiration capacity in the frozen PBMCs of 44 patients enrolled at the Metabolic Liver Diseases outpatient service at Fondazione IRCCS Cà Granda (Milan, Italy) who were affected by alpha1-antitrypsin deficiency (AAT; n = 16), hereditary hemochromatosis (HH; n = 16), alcoholic liver disease (ALD; n = 7) and autoimmune hepatitis (AH; n = 5) (MASLD-unrelated cohort). Diagnostic inclusion criteria included the presence of the PiZ allele in a homozygous or heterozygous state for AAT, the homozygosity for the C282Y mutation in the homeostatic iron regulator (HFE) gene for HH and alcohol intake (>30 g/day for males and >20 g/day for females) according to the ALD and EASL guidelines for AH [33]. Hepatic histology was assessed non-invasively using Fibroscan to estimate the presence of liver steatosis (CAP ≥ 248) and fibrosis (LSM ≥ 7.0 and ≥6.2 kPa) [34,35]. Informed written consent was obtained from each patient, and the study protocol was approved by the Ethical Committee of the Fondazione and conforms to the ethical guidelines of the 1975 Declaration of Helsinki. The demographic, anthropometric and clinical data of these patients are listed in Table S3. Patients belonging to the MASLD-unrelated cohort shared clinical features (sex, age and BMI) with MASLD individuals (Discovery cohort and Fibroscan-MASLD cohort) but differed in liver disease etiology.

### 2.4. Sample Collection, Storage and Homogenization

Frozen liver biopsies (1 to 3 mg) were manually homogenized in 250 μL of MAS buffer (70 mM sucrose, 220 mM mannitol, 5 mM KH_2_PO_4_, 5 mM MgCl_2_, 1 mM EGTA and 2 mM HEPES). Then, homogenates were centrifuged for 5 min at 1000× *g* and 4 °C. Collect the resulting supernatant was collected as the homogenate sample, and the pellet was discarded. The supernatant (tissue homogenate) may be stored at −80 °C for long-term storage.

A total of 300 μL of frozen whole blood was aliquoted in a new Eppendorf tube, and 900 μL of PBS 1X (1:3) was added, and the sample was centrifuged for 10 min at 100× *g* and 18–24 °C without break. The supernatant was centrifuged, and 2 mL of 1X PBS was added to resuspend the pellet of PBMCs. Then, the pellet was centrifuge at 100× *g* for 10 min at 18–24 °C without break; the supernatant was discarded, and the PBMC pellet was resuspended in 300 μL of MAS buffer. The PBMC homogenate may be stored at −80 °C for long-term storage. Both liver biopsies and PBMC homogenate in MAS buffer were quantified through the BCA assay.

### 2.5. Frozen Liver Tissues and PBMCs Respirometry: Seahorse Assay

We homogenized liver tissues and PBMCs in MAS buffer and performed a BCA assay to measure the total protein content from each sample. Next, we loaded 80 μg of liver biopsies and PBMCs in a Seahorse XF24 plate and added 20 μL of MAS buffer. The loaded plate was centrifuged for 5 min at 2000× *g* and 4 °C without brake using a plate carrier. A total of 350 μL of MAS buffer containing cytochrome c (10 μg/mL, final concentration) was added to allow for the complete membrane permeabilization of the substrates. The cartridge was prepared by injecting 70 μL of different drugs as follow: port A: NADH (1 mM) (Complex I substrate) for the complex I assessment or 5 mM succinate (Complex II substrate) + 2 μM rotenone (Complex I inhibitor) for the Complex II assessment; port B: antimycin A (4 μM) (Complex III inhibitor); port C: TMPD (electron donor to cytochrome c/Complex IV) + ascorbic acid (maintains TMPD in the reduced state) (0.5 mM TMPD + 1 mM ascorbic acid); port D: azide (Complex IV inhibitor) (50 mM). All compound injections should be diluted in MAS buffer. The measure times for the Seahorse run protocol was customized as follow: cycle 1: wait 2 min, mix 2 min and wait 2 min; measurement period 1: mix 50 s and measure 4 min (×4 cycles); injection 1 (port A) and measure after injection: mix 50 s and measure 4 min (×4 cycles); injection 2 (port B) and measure after injection: mix 50 s and measure 4 min (×4 cycles); injection 3 (port C) and measure after injection: mix 50 s and measure 4 min (×4 cycles); and injection 4 (port D) and measure after injection: mix 50 s and measure 4 min (×4 cycles). The maximal respiratory capacity was calculated as follow: through complex I: OCR_NADH_ − OCR_antimycin_, through complex II: OCR_succinate+rotenone_ − OCR_antimycin_ and through complex IV: OCR_TMPD+Ascorbate_ − OCR_azide_.

### 2.6. Statistical Analysis

Statistical analyses were performed using JMP16.0 (SAS, Cary, NC, USA), R statistical analysis version 3.3.2 and Prism (V.9, San Diego, CA, USA), using one-way analysis of variance (ANOVA) or the chi-square test where appropriate. For descriptive statistics, continuous variables are shown as the mean and standard deviation or the median and interquartile range for highly skewed biological variables (i.e., ALT). Variables with skewed distributions were logarithmically transformed before analyses. Categorical variables are presented as numbers and proportions. Analyses were performed by fitting the data to generalized the linear regression models. Generalized linear models were fitted to examine continuous traits. Multinomial logistic regression models were fitted to examine binary traits, and ordinal regression models were fitted for ordinal traits (components of the MASLD activity score: severity of steatosis, necroinflammation and hepatocellular ballooning and stage of fibrosis). When specified, confounding factors were included in the model. For gene expression analyses, differences between groups were calculated using one-way ANOVA, which was followed by post hoc t-tests adjusted for the number of comparisons when multiple groups were involved (Bonferroni correction). *p*-values < 0.05 (two-tailed) were considered statistically significant.

## 3. Results

### 3.1. Restoring MBOAT7 and/or TM6SF2 WT Activities in KO Models Rebalances the Mitobiogenesis Process

It is well established that the impaired mitochondrial number and architecture result from unbalanced mitobiogenesis and flow towards maladaptive organelle activity. We recently demonstrated that the *PNPLA3*, *MBOAT7* and *TM6SF2* loss-of-function mutations affect the mitochondrial morphology and biomass in hepatocytes, although we did not provide any data regarding their impact on the organelle’s dynamics [23]. Therefore, in this study, we firstly investigated the mechanisms through which the deletion of these genes may impair the mitochondrial lifecycle by exploiting HepG2 *MBOAT7^−/−^*, *TM6SF2^−/−^* and *MBOAT7^−/−^TM6SF2^−/−^* in vitro models.

Then, we restored the WT proteins through lentiviral transfection in KO cells to obtain stable cell lines that overexpress the MBOAT7 (*MBOAT7^+/+^*) and/or *TM6SF2* (*TM6SF2^+/+^*; *MBOAT7^+/+^ TM6SF2^+/+^*) genes (Figure S1A–F) and to assess whether mitochondrial dynamics, maladaptive function, metabolic reprogramming and advanced injury may be reversed.

We previously demonstrated that the silencing of the *MBOAT7* and *TM6SF2* genes in HepG2 cells (homozygous for the PNPLA3 I148M mutation) triggered fat accumulation. As evidence that the activity of MBOAT7 and TM6SF2 had been restored, lipid overload, which was evaluated via ORO staining (Figure S1G,H) and triglyceride (Figure S1I)/cholesterol (Figure S1J) content, was reduced in *MBOAT7^+/+^*, *TM6SF2^+/+^* and *MBOAT7^+/+^TM6SF2^+/+^* cells (Figure S1G–J).

**Figure 1:**
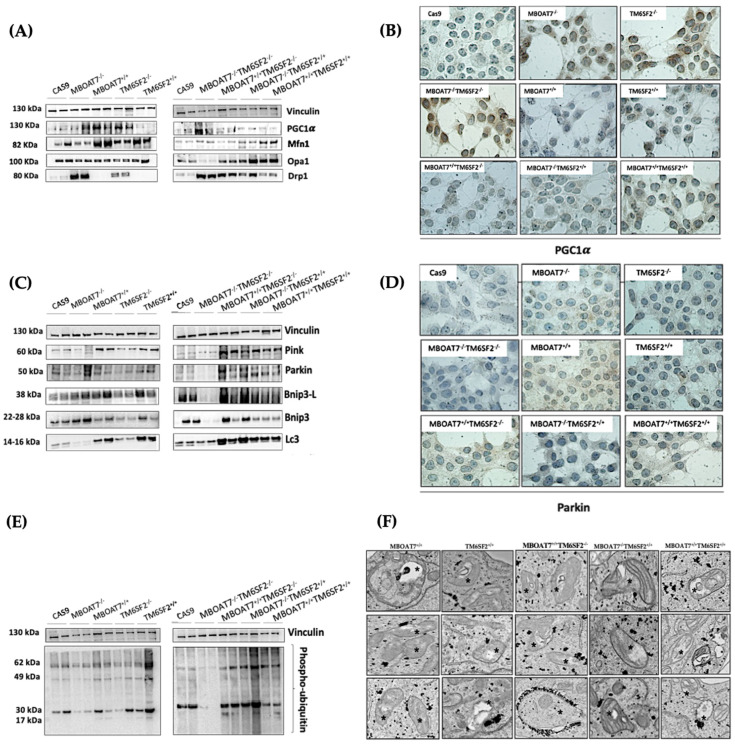
*MBOAT7* and/or *TM6SF2* WT overexpression rebalances the mitochondrial lifecycle and turnover. (**A**,**C**,**E**) The protein levels of PGC1α, Mfn1, Opa1, Drp1, Pink, Parkin, Bnip3-L, Bnip3, LC3 and phopsho-ubiquitin were assessed using Western blotting and normalized to the vinculin housekeeping gene. (**B**–**D**) The cytoplasmatic and nuclear localization of PGC1α and Parkin were represented using immunocytochemistry pictures (magnification 100×). (**F**) Representative TEM images of autophagic structures in the form of vacuoles were obtained using ultrathin 70 nm sections of hepatocytes (scale bar 0.5 μm). Black stars indicate the autophagic structures in the form of vacuoles in mitochondria and cytoplasm. At least 3 independent experiments were conducted. For bar graphs, data are expressed as means and SD.

**Figure 2:**
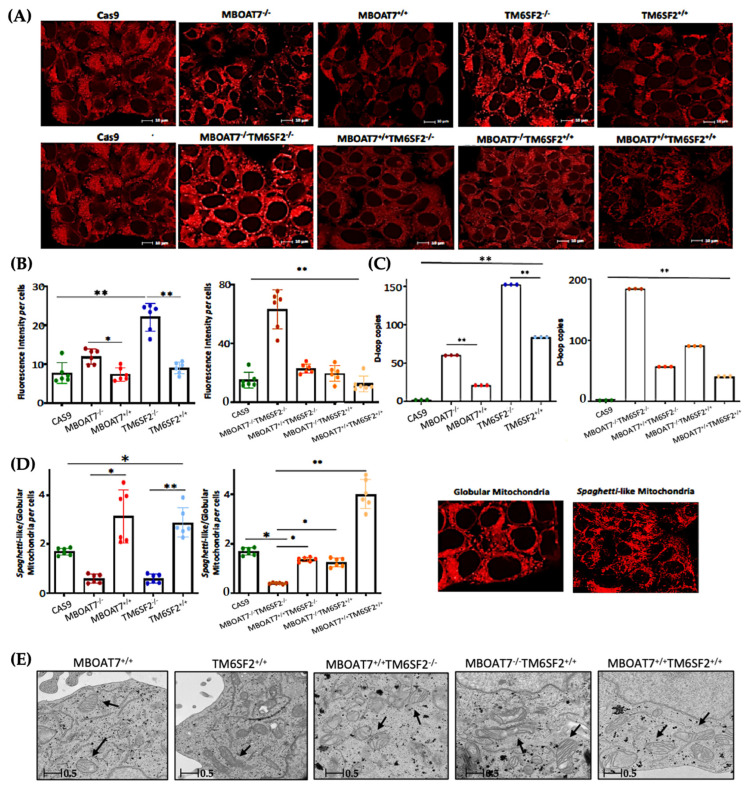
The WT upregulation of the *MBOAT7* and/or *TM6SF2* WT genes in knock-out models resumes the assembly of physiologically shaped mitochondria. (**A**) Representative confocal microscopy images (size range: 10 μm) of mitochondria were obtained using Mito Tracker staining (magnification 60×). (**B**) The fluorescence intensity per cells was quantified using ImageJ (1.48v) in 6 random nonoverlapping micrographs per condition by calculating the percentage of pixels above the threshold value with respect to total pixels per area. (**C**) D-loop copies that disclose the intracellular number of mitochondria were calculated using reverse-transcription quantitative PCR and normalized to the RnaseP housekeeping gene. (**D**) The ratio between spaghetti-like and globular-shaped mitochondria per cells was quantified using ImageJ in 6 random nonoverlapping micrographs per condition by calculating the percentage of pixels above the threshold values of spaghetti-like and globular-shaped mitochondria, respectively, with respect to total pixels per area (magnification 60×). (**E**) Representative TEM images of mitochondria were obtained using ultrathin 70 nm sections of hepatocytes. Black arrows indicate the mitochondria (scale bar 0.5 μm). At least 3 independent experiments were conducted. For bar graphs, data are expressed as means and SD. Adjusted * *p* < 0.05 and ** *p* < 0.01.

Next, we went on in evaluating mitochondrial dynamics in KO and overexpressed cells. The protein (Figure 1A,B) and mRNA (Figure S2A) levels of PGC1α, a key regulator of the mitochondrial lifecycle and β-oxidation, increased in *MBOAT7^−/−^* cells and much more in *TM6SF2^−/−^* and *MBOAT7^−/−^TM6SF2^−/−^* ones (adjusted ** (adj-*p*) < 0.01 and * adj-*p* < 0.05 vs. Cas9) as a counter strategy in response to lipid overload (Figure S1G–J). In keeping with reduced lipid accumulation, the replenishment of WT MBOAT7 and TM6SF2 proteins declined PGC1α levels, showing the strongest effect in the *MBOAT7^+/+^TM6SF2^+/+^* cell line (Figure 1A,B and Figure S2A: ** adj-*p* < 0.01 vs. *MBOAT7^−/−^* and *TM6SF2^−/−^*; * adj-*p* < 0.05 vs. *MBOAT7^−/−^TM6SF2^−/−^*). Concerning mitochondrial fusion, Mfn1 (Figure 1A and Figure S2B–F), Mfn2 (Figure S2C–G) and Opa1 (Figure 1A and Figure S2H) levels were lower in KO cells and mostly in the *MBOAT7^−/−^TM6SF2^−/−^* model, whereas they were rescued in WT cells and especially in *MBOAT7^+/+^TM6SF2^+/+^* ones (* adj-*p* < 0.05 vs. *MBOAT7^−/−^*, *TM6SF2^−/−^* and *MBOAT7^−/−^TM6SF2^−/−^*). The mRNA expression and protein activity of DRP1 (Figure 1A and Figure S2D–I) and Fis1 (Figure 1A and Figure S2E), which are involved in mitochondrial fission, were higher in *MBOAT7^−/−^TM6SF2^−/−^* cells, while they returned under physiological conditions in WT overexpressed models (* adj-*p* < 0.05 vs. Cas9 and *MBOAT7^−/−^TM6SF2^−/−^*; ** adj-*p* < 0.01 vs. *MBOAT7^−/−^*, *TM6SF2^−/−^* and *MBOAT7^−/−^TM6SF2^−/−^*). Finally, the silencing of *MBOAT7* and *TM6SF2* alone, as well as in combination, repressed the mitophagy pathway exhibiting vacuolar autophagic structures, which were recovered in WT cells and especially in *MBOAT7^+/+^TM6SF2^+/+^* clones (Figure 1C–F). Taken together, these data suggest that the deletion of both *MBOAT7* and *TM6SF2* triggers an imbalance of the mitochondrial lifecycle towards high fission and non-operative mitophagy, which explains the increased mass of misfolded organelles in the KO models, as confirmed using confocal microscopy (Figure 2A,B). Moreover, we observed that the D-loop copies, reflecting mitochondrial mass, were increased in the silenced models (Figure 2C). Conversely, the total number of organelles as well as D-loop copies was lower in the overexpressed models and especially in *MBOAT7^+/+^TM6SF2^+/+^* clones (** adj-*p* < 0.01 vs. Cas9, *MBOAT7^−/−^*, *TM6SF2^−/−^*, *MBOAT7^−/−^TM6SF2^−/−^*) (Figure 2A–C), thus suggesting that the recovery of WT proteins resets mitobiogenesis, possibly ensuring the assembly of physiological mitochondria (referred to as *spaghetti*-like).

### 3.2. Mitochondrial Morphology Is Restored by the Expression of Wild-Type MBOAT7 and/or TM6SF2 Proteins in Knock-Out Models

The aberrant bulk of mitochondria is paralleled by an altered morphology. Indeed, we pointed out that KO cells exhibited many misshaped globular mitochondria compared to the physiological *spaghetti*-like morphology observed in WT overexpressed models (Figure 2D), supporting that *TM6SF2* and *MBOAT7* deletion led to deranged mitobiogenesis paralleled by suppressed mitophagy (Figure 1 and Figure S2) (* adj-*p* < 0.05 vs. Cas9, *MBOAT7^−/−^* and *MBOAT7^−/−^TM6SF2^−/−^*; ** adj-*p* < 0.01 vs. *TM6SF2^−/−^* and *MBOAT7^−/−^TM6SF2^−/−^*). Consistent with the re-established mitochondrial plasticity in WT overexpressed cells, the organelles appeared more elongated and showed regular double membranes and a cristae architecture (Figure 2E), thus reinforcing the hypothesis according to which the higher number of mitochondria may be related to an imbalance in the lifecycle rather than to enhanced organelles’ function.

### 3.3. The Overexpression of WT MBOAT7 and/or TM6SF2 Proteins in KO Cells Rescues Mitochondrial Function

**Figure 3:**
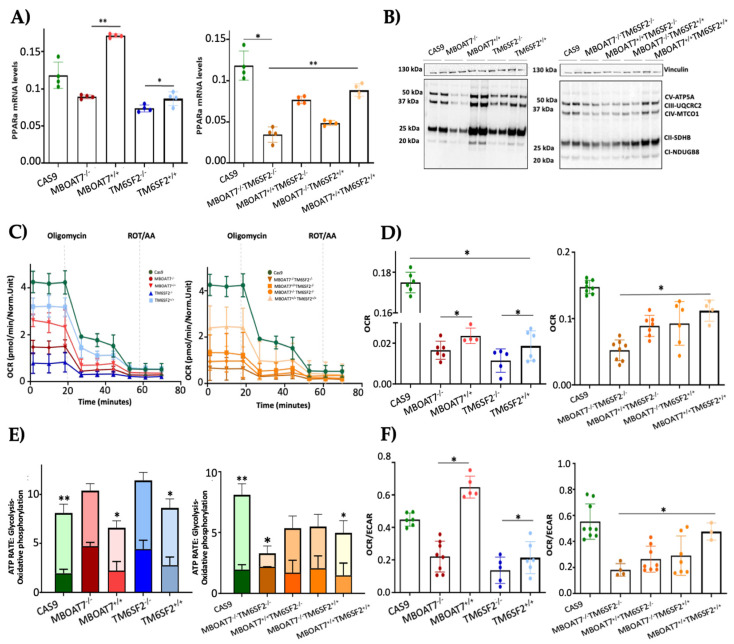
WT *MBOAT7* and/or *TM6SF2* overexpression in knock-out models restores mitochondrial functions. (**A**) The mRNA expression of *PPAR*α was evaluated using reverse- transcription quantitative PCR and normalized to the β-actin housekeeping gene. (**B**) The protein levels of OXPHOS complexes I, II, III, IV and V were assessed using Western blotting and normalized to the vinculin housekeeping gene. (**C**,**D**) The oxygen consumption rate (OCR) was obtained via the Mito Stress test using the Seahorse XF Analyzers in live cells. (**E**,**F**) The ATP rate derived from glycolysis and oxidative phosphorylation and the OCR/extracellular acidification rate (ECAR) ratio were measured using a glycolytic assay by exploiting the Seahorse XF Analyzers in live cells. At least 3 independent experiments were conducted. For bar graphs, data are expressed as means and SD. Adjusted * *p* < 0.05 and ** *p* < 0.01.

To assess whether the overexpression of *MBOAT7* and *TM6SF2* rescues organelles’ function, we firstly evaluated the mRNA levels of *PPAR*α (Figure 3A), a master regulator of FFA oxidation. The overexpressed cell lines showed higher expression of *PPAR*α, thus demonstrating that the steatotic phenotype was attenuated after MBOAT7 and/or TM6SF2 rescue (Figure S1). Concerning mitochondrial respiration and the Krebs cycle, the OXPHOS capacity (Figure 3B) and the activity of complexes III and IV, ATP synthase and citrate synthase (Figure S3A–D) were decreased in KO cells and mainly in *MBOAT7^−/−^TM6SF2^−/−^* ones, in keeping with low mitochondrial fusion (Figure 1A and Figure S2B,C,F–H). Conversely, their levels were restored in WT overexpressed models especially in the *MBOAT7^+/+^TM6SF2^+/+^* clone, in agreement with a balanced mitobiogenesis (Figure 1 and Figure S2) and physiological mitochondrial plasticity (Figure 2) (** adj-*p* < 0.01 vs. Cas9 and *MBOAT7^−/−^TM6SF2^−/−^*; * adj-*p* < 0.05 vs. Cas9, *TM6SF2^−/−^* and *MBOAT7^−/−^TM6SF2^−/−^*). In keeping with the altered mitochondrial architecture and respiration capacity, we observed reduced ATP production and reduced mitochondrial membrane potential in *MBOAT7^−/−^* and *TM6SF2^−/−^* and more so in *MBOAT7^−/−^TM6SF2^−/−^* cell lines, resulting in the opening of the mitochondrial permeability transition pores and leading to a higher release of calcium (Figure S3E–G). Notably, low levels of calcium are essential to maintain the optimal rates of ATP production, whereas an elevation of calcium release leads to the loss of mitochondrial function and changes in the mitochondria ultrastructure and cristae network [36]. Conversely, restoring WT MBOAT7 and/or TM6SF2 activities rescued the ATP levels and rebalanced the mitochondrial membrane potential alongside the efflux of calcium (Figure S3E–G).

The quantitative measurement of the total ATP rate derived from mitochondrial and glycolytic pathways revealed that the mitochondrial oxygen consumption rate (OCR), which was lower in the *MBOAT7^−/−^TM6SF2^−/−^* clone, increased in WT overexpressed models and mostly in *MBOAT7^+/+^TM6SF2^+/+^* cells (Figure 3C,D) (* adj-*p* < 0.05 vs. Cas9, *MBOAT7^−/−^*, *TM6SF2^−/−^* and *MBOAT7^−/−^TM6SF2^−/−^*). Consistently, ATP production derived equally from the glycolytic pathway and mitochondria in all KO cell lines except for the *MBOAT7^−/−^TM6SF2^−/−^* one, which showed the highest extracellular acidification rate (ECAR) as the energy source from anaerobic glycolysis (Figure 3E,F) (** adj-*p* < 0.001, * adj-*p* < 0.05 vs. own ATP glycolysis). Contrarywise, the overexpression of *MBOAT7* and *TM6SF2* alone and mainly combined enhanced ATP production related to OXPHOS capacity, thus recovering mitochondrial functions and impeding metabolic reprogramming, which is a hallmark of tumorigenesis (Figure S4).

### 3.4. The Restoration of WT MBOAT7 and/or TM6SF2 Proteins in KO Cells Attenuates Hepatocellular Damage

**Figure 4:**
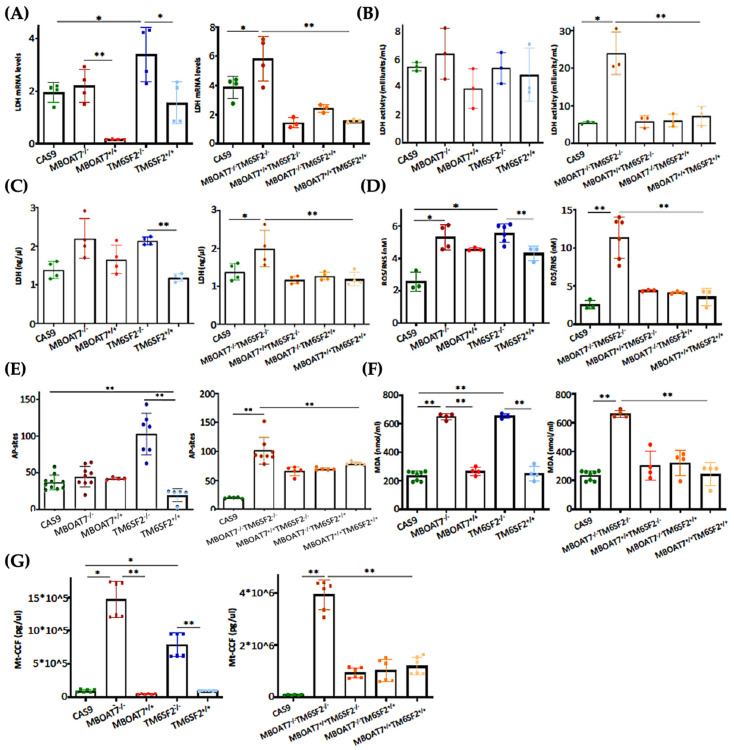
WT *MBOAT7* and/or *TM6SF2* upregulation in knock-out cells attenuates hepatocellular injury. (**A**) The mRNA expression of *LDH* was evaluated using reverse-transcription quantitative PCR and normalized to the β-actin housekeeping gene (**B**) LDH activity was measured using the Lactate Dehydrogenase (LDH) Assay Kit in cell lysates. (**C**) LDH production was quantified through LDH activity using Lactate Dehydrogenase (LDH) Assay Kit in cell supernatants. (**D**) Oxidative stress was evaluated using the Dichlorodihydrofluorescin (DCF) reactive oxygen species/reactive nitrogen species (ROS/RNS) Colorimetric Assay Kit in cell lysates. (**E**) ROS-induced DNA damage was detected through the DNA Damage Colorimetric Assay Kit (apurinic/apyrimidinic (AP) sites) in cell lysates. (**F**) Malondialdehyde (MDA) production was calculated using the Lipid Peroxidation (MDA) Assay Kit in cell lysates. (**G**) The release of cell-free mitochondrial DNA fragments (mt-ccf) was quantified through quantitative real-time PCR and normalized on the standard curve obtained from serial dilutions of a sample pool at known concentrations. At least 3 independent experiments were conducted. For bar graphs, data are expressed as means and SD. Adjusted * *p* < 0.05 and ** *p* < 0.01.

The switch toward anaerobic glycolysis (Figure 3 and Figure S3) is one of the first key steps in malignant transformation paralleled by high proliferation capacity and invasiveness (Figure S4). In support of this notion, KO models and particularly the *MBOAT7^−/−^TM6SF2^−/−^* clone converted glucose into lactate by triggering Lactate Dehydrogenase (LDH) activity and production, both of which are reduced in WT overexpressed models (Figure 4A–C) (* adj-*p* < 0.05 vs. Cas9 and TM6SF2^−/−^; ** adj-*p* < 0.01 vs. *MBOAT7^−/−^* and *MBOAT7^−/−^TM6SF2^−/−^*). Lactate directly takes part in oxidative damage, resulting in a boosted reactive oxygen species (ROS)/reactive nitrite species (ROS/RNS) ratio, ROS-induced DNA damage and malondialdehyde (MDA) production, which were higher mostly in *MBOAT7^−/−^TM6SF2^−/−^* cells, whereas they strongly decreased after WT *MBOAT7* and/or *TM6SF2* upregulation (Figure 4D–F) (* adj-*p* < 0.05 and ** adj-*p* < 0.01 vs. Cas9, *MBOAT7^−/−^*, *TM6SF2^−/−^* and *MBOAT7^−/−^TM6SF2^−/−^*). As a response to unbalanced mitobiogenesis (Figure 1 and Figure S2) and failed mitochondrial activity (Figure 3 and Figure S3), KO models released higher circulating cell-free mitochondrial DNA (ccf-mtDNA) derived from damaged organelles (Figure 4G). Otherwise, the upregulation of WT MBOAT7 and/or TM6SF2 proteins greatly reduced the release of ccf-mtDNA (Figure 4G), corroborating the hypothesis that the high number of mitochondria is not representative of an enhanced function and that genetics directly impacts the integrity of mitochondria. Notably, ccf-mtDNA correlated with the lower activity of complex V (ATP synthase) (*p* < 0.0001, β = −1.1 and 95%CI: −1.56–−0.64) and reduced OCR (*p* < 0.0001, β = −9.9 and 95%CI: −16.77–−3.10). Hence, the assessment of ccf-mtDNA levels may mirror mitochondrial derangement due to *TM6SF2* and *MBOAT7* deletion, thus paving the way to consider them as a mitochondrial circulating biomarker of progressive MASLD in genetically predisposed individuals.

### 3.5. PNPLA3 I148M Overexpression in Hepatoma Cells Impairs Mitochondrial Function

In our MASLD in vitro models, we deeply demonstrated that the MBOAT7 and TM6SF2 loss-of-function mutations in HepG2 cells triggered mitochondrial maladaptation, exhibiting the strongest effect when both genes were deleted (*MBOAT7^−/−^TM6SF2^−/−^*). Moreover, the restoration of WT MBOAT7 and TM6SF2 activities rescued the mitochondrial phenotype, corroborating their role in contributing to mitochondrial dysfunction encompassing lifecycle, morphology and activity.

**Figure 5:**
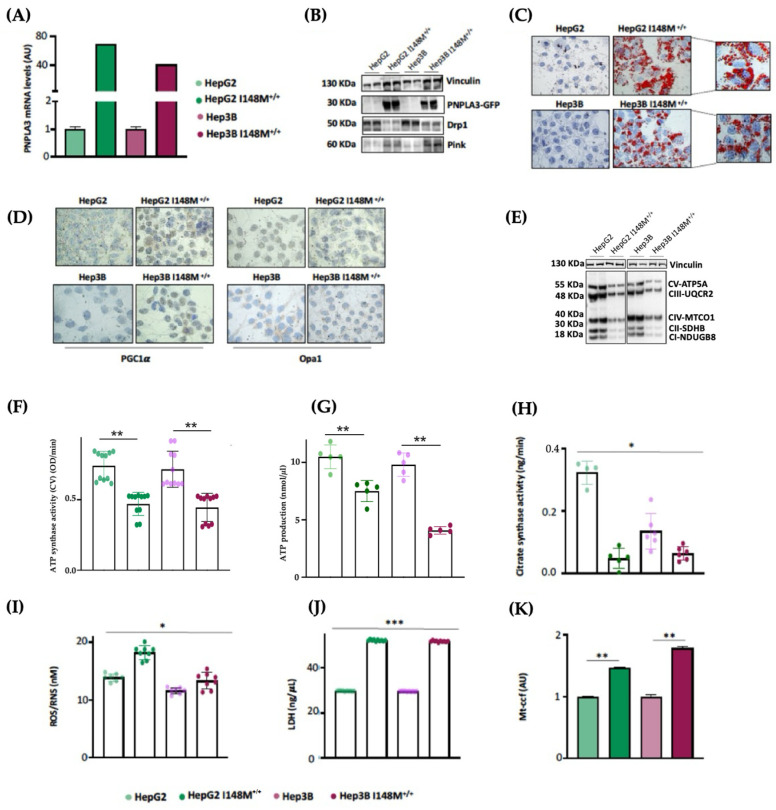
I148M^+/+^ PNPLA3 upregulation in hepatoma cells triggers lipid accumulation alongside mitochondrial failure. (**A**) The mRNA expression of *PNPLA3* was evaluated using reverse-transcription quantitative PCR and normalized to the β-actin housekeeping gene. (**B**) The protein levels of PNPLA3 tagged with GFP (PNPLA3-GFP) were assessed using Western blotting and normalized to the vinculin housekeeping gene. (**C**) LD accumulation was assessed via ORO staining (magnification 630×). (**D**) The cytoplasmatic and nuclear localization of PGC1α and Opa1 were represented using immunocytochemistry pictures (magnification 100×). (**E**) The protein levels of OXPHOS complexes I, II, III, IV and V were assessed using Western blotting and normalized to the vinculin housekeeping gene. (**F**) ATP5A (mtDNA-encoded subunit of complex V) activity was quantified using the ATP Synthase Enzyme Activity Microplate Assay Kit in isolated mitochondria from cell lysates. (**G**) ATP production was quantified using the ATP Colorimetric Assay in cell lysates. (**H**) Citrate synthase activity was assessed through the Citrate Synthase Assay Kit in isolated mitochondria from cell lysates. (**I**) ROS-induced DNA damage was detected through the DNA Damage Colorimetric Assay Kit (apurinic/apyrimidinic (AP) sites) in cell lysates. (**J**) LDH production was quantified through LDH activity using the Lactate Dehydrogenase (LDH) Assay Kit in cell supernatants. (**K**) The release of cell-free mitochondrial DNA fragments (mt-ccf) was quantified through quantitative real-time PCR and normalized on the standard curve obtained from serial dilutions of a sample pool at known concentrations. At least 3 independent experiments were conducted. For bar graphs, data are expressed as means and SD. Adjusted * *p* < 0.05, ** *p* < 0.01 and *** *p* < 0.001.

As HepG2 cells carry the PNPLA3-I148M variant in homozygosity, we were not able to discriminate its effect on mitochondrial morphology and function from that of TM6SF2 and MBOAT7 in KO models. Interestingly, Cas9 cells carrying the I148M protein did not affect the organelles dynamics and function, hinting that the PNPLA3 loss-of-function mutation may not play a role in mitochondrial dysfunction. Nonetheless, evidence from the literature showed that the overexpression of I148M in hepatoma cells was correlated with mitochondrial impairment [27]. Therefore, to clarify the impact of PNPLA3 variation on mitochondrial dynamics, we performed lentiviral transfection in HepG2 (in order to force the mutation effect) and Hep3B (which are WT for the PNPLA3-I148M mutation) cells by using pLenti-C-mGFP-P2A-Puro lentiviral vectors (HepG2 I148M^+/+^ and Hep3B I148M^+/+^). The vector design embraced a GFP tag fused with an ORF (PNPLA3-I148M) targeting the I148M protein that was upregulated in all cell lines (Figure 5A,B) (** adj-*p* < 0.01 vs. HepG2 and Hep3B). In terms of ORO staining, lipid accumulation increased in Hep3B I148M^+/+^ cells and more so in HepG2 I148M^+/+^ ones, resulting in the assembly of larger LDs (Figure 5C). Concerning mitobiogenesis, PGC1α protein levels augmented in both overexpressed models, suggesting a responsive strategy to counter fat accumulation (Figure 5D). Regarding fusion, Opa1 activity was reduced in the overexpressed cell lines; this was (Figure 5D) supported by lower OXPHOS capacity observed in the Western blot (Figure 5E). In keeping with these results, ATP synthase activity, ATP content and citrate synthase activity decreased in all overexpressed cells, emphasizing that I148M overexpression dampened oxidative phosphorylation, probably due to high lipid overload (Figure 5F–H) (*** adj-*p* < 0.001, ** adj-*p* < 0.01 and * adj-*p* < 0.05 vs. HepG2 and Hep3B). Consistently, the ROS/RNS ratio (Figure 5I) and LDH production (Figure 5J) were boosted in cells overexpressing the mutated protein alongside the release of ccf-mtDNA (Figure 5K) (*** adj-*p* < 0.001, ** adj-*p* < 0.01 and * adj-*p* < 0.05 vs. HepG2 and Hep3B). To sum up, the upregulation of the I148M-PNPLA3 protein in in vitro hepatoma cells by resembling the overload of the mutated protein in human carriers unveiled its involvement in mitochondrial dysfunction.

### 3.6. Hepatic and Circulating Mitochondrial Activity Is Impaired in MASLD Patients Carrying the Three At-Risk Variants

**Figure 6:**
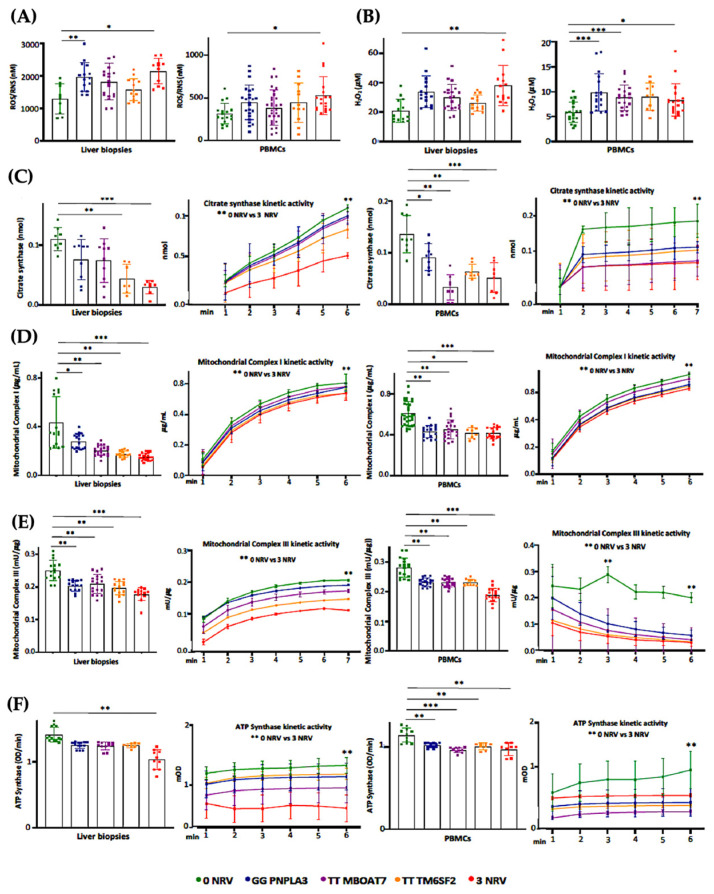
Mitochondrial activity is impaired in the liver biopsies and PBMCs of 3NRV MASLD carriers. (**A**,**B**) Oxidative stress was evaluated using the Dichlorodihydrofluorescin (DCF) reactive oxygen species/reactive nitrogen species (ROS/RNS) Colorimetric Assay Kit in frozen liver biopsies and PBMCs. (**C**,**D**) Complex I/III enzymatic activity was measured through a colorimetric assay in frozen liver biopsies and PBMCs. (**E**) ATP5A (mtDNA-encoded subunit of the complex V) activity was quantified using the ATP Synthase Enzyme Activity Microplate Assay Kit in frozen liver biopsies and PBMCs (**F**). At least 3 independent experiments were conducted. For bar graphs, data are expressed as means and SD. Adjusted * *p* < 0.05, ** *p* < 0.01 and *** *p* < 0.001.

Based on our in vitro results, the co-presence of the *PNPLA3*, *MBOAT7* and *TM6SF2* loss-of-function mutations in HepG2 cells impacts mitochondrial plasticity by exhibiting the restoration of organelles’ dynamics and function after the overexpression of WT proteins. Mitochondrial impairment is mirrored by the release of ccf-mtDNA. All in all, these genes have been identified as the main predictors of progressive MASLD which is featured by damaged mitochondria which in turn release ccf-mtDNA. Therefore, in order to translate these in vitro findings to clinical settings and to consider the assessment of mitochondrial molecules as non-invasive biomarkers, we evaluated and compared mitochondrial activity in frozen liver biopsies and peripheral blood mononuclear cells (PBMCs) in 44 MASLD patients (Discovery cohort). Patients were stratified according to their genetic background as follows: WT (n = 11; 22.7%), homozygous for I148M PNPLA3 (n = 11; 25.0%), homozygous for rs641738 MBOAT7 (n = 9, 20.5%), homozygous for E167K TM6SF2 (n = 7, 15.9%) and carrying all three at-risk variants (3NRV) in heterozygosis or homozygosis (n = 7, 15.9%) (Table S1). Hepatic ROS (Figure 6A, Table S6) and H_2_O_2_ (Figure 6B, Table S6) levels increased in GG *PNPLA3*, TT *MBOAT7* and TT *TM6SF2* carriers and even more in 3NRV ones (* *p* < 0.05 and ** *p* < 0.001 vs. 0NRV). Interestingly, a comparable result was observed in PBMC homogenates, suggesting that their mitochondrial performance may reflect the hepatic one (Figure 6A,B). Concerning mitochondrial respiration, the total and kinetic activities of citrate synthase (Figure 6C and Tables S6 and S7), mitochondrial complex I (Figure 6D and Tables S6 and S7), mitochondrial complex III (Figure 6E and Tables S6 and S7) and ATP synthase (Figure 6F and Tables S6 and S7) were lower in both liver biopsies and PBMCs of GG *PNPLA3*, TT *MBOAT7* and TT *TM6SF2* individuals showing the main reduction in 3NRV carriers (* *p* < 0.05, ** *p* < 0.001, *** *p* < 0.0001 vs. 0NRV). Therefore, we firstly demonstrated that in genetically susceptible MASLD patients, mitochondrial failure in the PMBC closely mirrors the hepatic one.

### 3.7. Serum Bioenergetic Profile Resembles the Hepatic One and Prognoses MASLD Severity in 3NRV Carriers

**Figure 7:**
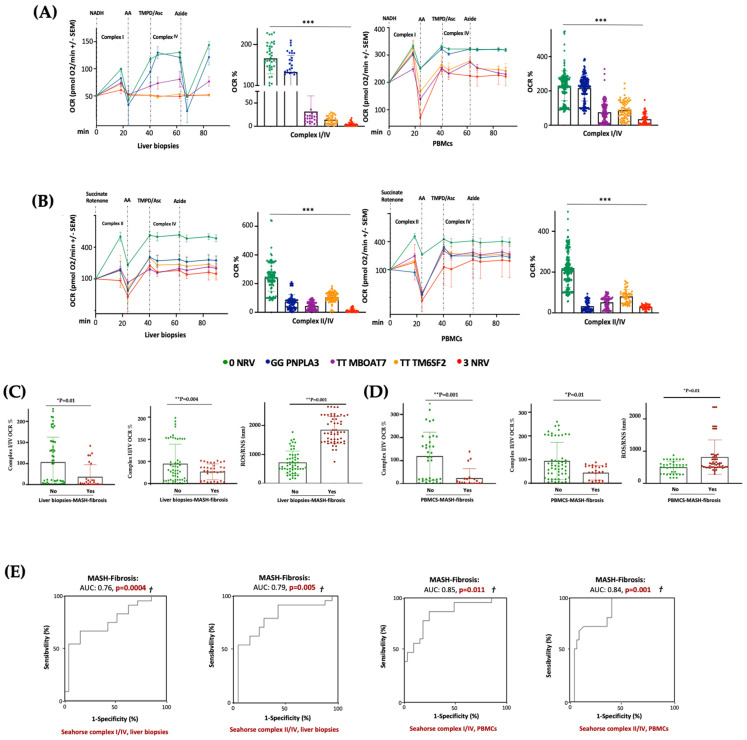
The PBMC bioenergetic profile reflects the hepatic one and exhibits a strong prognostic value for MASH-Fibrosis in 3NRV carriers. (**A**,**B**) The oxygen consumption rate (OCR) was obtained through a customized Seahorse assay using the Seahorse XF Analyzers in frozen hepatic biopsies and PBMCs. At least 4 independent experiments were conducted. For bar graphs, data are expressed as means and SD. Adjusted * *p* < 0.05, ** *p* < 0.01 and *** *p* < 0.001. (**C**,**D**) According to a bivariate analysis, patients with MASH-fibrosis exhibit lower OCR and high ROS production (Liver biopsies of complex I/IV: *p* = 0.01; Liver biopsies of complex II/IV: *p* = 0.004; Liver biopsies for ROS/RNS: *p* = 0.001; PBMCs’ complex I/IV: *p* = 0.001; PBMCs’ complex II/IV: *p* = 0.01; PBMCs’ ROS/RNS: *p* = 0.01). (**E**) The multinomial logistic regression analysis adjusted for 3NRV show that complexes I/IV and II/IV have a strong prognostic accuracy for MASH-Fibrosis (Liver biopsies of complex I/IV: AUC = 0.76 and † *p* = 0.0004; Liver biopsies of complex II/IV: AUC = 0.79 and † *p* = 0.005; PBMCs’ complex I/IV: AUC = 0.85 and † *p* = 0.011; PBMCs’ complex II/IV: AUC = 0.84 and † *p* = 0.001).

In keeping with the impaired mitochondrial activity, we demonstrated that hepatic and sera OCR progressively decreased from I148M PNPLA3 carriers (30%) to rs641738 MBOAT7 (70%) or E167K TM6SF2 (80%) variations, with the lowest levels in 3NRV individuals (90%) (*** *p* < 0.0001 vs. 0NRV) (Figure 7A,B), thus resembling the declined OXPHOS capacity observed in the *MBOAT7^−/−^TM6SF2^−/−^* model (Figure 3 and Figure S3). According to the multivariable analysis adjusted for age, sex, BMI and diabetes, the co-presence of 3NRV was independently associated with reduced respiration in terms of complex I/IV (Table S8. Liver biopsies: *p* = 0.0001, β = −43.37, and 95%CI: −64.81–−21.92; PBMCs: *p* = 0.002, β = −30.7 and 95%CI: −49.96–−11.45) and II/IV in both hepatic samples and sera (Table S9. Liver biopsies: *p* = 0.0001, β = −27.24 and 95%CI: −10.47–−14.02; PBMCs: *p* < 0.0001, β =−20.08 and 95%CI: −25.20–−14.96). These data highlight that genetics directly compromises the circulating bioenergetic profile which closely reflects hepatic mitochondrial respiration. To investigate whether impaired OCR could estimate mitochondrial failure and thereby worsen the disease, patients belonging to the Discovery cohort were stratified according to the presence of MASH-fibrosis. The liver biopsies and PBMC samples of MASH-Fibrosis patients exhibited a significant OCR reduction in terms of complex I/IV and II/IV activities and a subsequent increase in ROS/RNS production (Figure 7C,D) (* *p* and ** *p* according to two-way ANOVA), both of which are features of worsened damage. According to the multinomial logistic regression analysis adjusted for 3NRV, the hepatic respiration capacity of complexes I/IV and II/IV displayed a prognostic value for MASH-Fibrosis of 76% and 79%, respectively (Figure 7D). Intriguingly, the sera of OCR-I/IV and OCR-II/IV revealed a competitive prediction capacity for MASH-Fibrosis of 85% and 84% (Figure 7E). Therefore, we confirmed that circulating mitochondrial respiration reflects that observed in the liver, thus making reasonable the applicability of mitochondrial molecules (i.e., ccf-mtDNA) as non-invasive biomarkers of advanced liver disease in patients with a genetic predisposition.

### 3.8. Serum Bioenergetics Predicts Fibrosis in Non-Invasively Assessed MASLD Patients Carrying 3NRV

**Figure 8:**
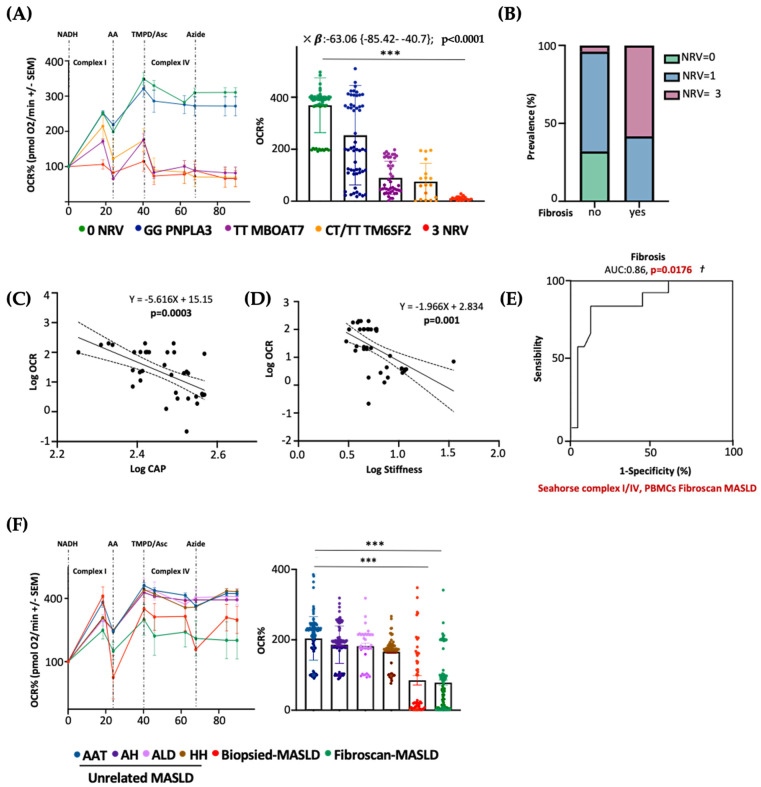
The PBMC bioenergetic profile predicts fibrosis in both non-invasively and biopsied MASLD patients carrying the three at-risk variants. (**A**) The oxygen consumption rate (OCR) was obtained through a customized Seahorse assay using the Seahorse XF Analyzers in frozen PBMCs. At least 4 independent experiments were conducted. For bar graphs, data are expressed as means and SD. Adjusted *** *p* < 0.001. According to the multivariable analysis adjusted for age, sex, BMI and diabetes, the co-presence of 3NRV was independently associated with reduced respiration (X: *p* < 0.0001, β = −63.06, and 95%CI: −85.42 to −40.7). (**B**) Patients were stratified according to their Fibroscan evaluation as fibrosis no = stiffness < 7 kPa (n = 30) and fibrosis yes = stiffness > 7 kPa (n = 15). Based on genetic variations and histological assessments, the subjects were grouped as follow: NRV = 0, fibrosis, no (n = 9, 100%); NRV = 1, fibrosis, no (n = 20, 76.19%), fibrosis yes (n = 6, 23.81%); NRV = 3, fibrosis, no (n = 1, 12.50%), fibrosis yes (n = 9, 87.50%). The prevalence (%) was calculated based on the total number of patients/group. (**C**,**D**) Linear correlation analyses of liver steatosis (CAP) and fibrosis (stiffness) scores with the oxygen consumption rate (OCR). Overall, the Fibroscan-MASLD patients were included, and the simple linear regression equation and corresponding *p* value were shown in the figures. (**E**) Complexes I/IV have a strong prognostic accuracy for fibrosis (stiffness > 7 kPa) (PBMCs’ complex I/IV: AUC = 0.86 and † *p* = 0.0176) according to the multinomial logistic regression analysis adjusted for 3NRV. (**F**) The oxygen consumption rate (OCR) was obtained through a customized Seahorse assay using the Seahorse XF Analyzers in frozen PBMCs. At least 4 independent experiments were conducted. For bar graphs, data are expressed as means and SD. Adjusted *** *p* < 0.001.

Next, we evaluated whether PBMC mitochondrial respiration may also predict advanced diseases in patients with a non-invasive diagnosis of MASLD. To this purpose, we measured serum OCR in 45 MASLD patients included in the Fibroscan-MASLD cohort. PBMC respiration hugely declined in rs641738 MBOAT7 and E167K TM6SF2 carriers and mainly in 3NRV individuals (*** *p* < 0.0001 vs. 0NRV) (Figure 8A), thus mirroring the OCR genetic-based signature observed in biopsied patients (Discovery cohort) (Figure 7B). According to the multivariable analysis adjusted for age, sex, BMI and diabetes, the co-presence of 3NRV was independently associated with reduced serum respiration (Table S10. *p* < *0*.0001, β = −63.06, and 95%CI: −85.42–−40.7), corroborating the direct role of genetics in impairing PBMC respirometry. To validate that the altered circulating OCR could estimate advanced hepatic diseases, these patients were stratified according to the presence of fibrosis (stiffness > 7 kPa). The latter was higher in 3NRV carriers (n = 9, 87.50%) than in 0NRV (n = 0, 0%) and 1NRV (n = 6, 23.81%) (Figure 8B).

Moreover, serum OCR inversely correlated with CAP–steatosis and stiffness–fibrosis scores (Figure 8C: Log OCR vs. Log CAP *p* = 0.0003; Figure 8D: Log OCR vs. Log stiffness *p* = 0.001), exhibiting a strong prognostic value for fibrosis in the ROC curve (86%) (Figure 8E) and confirming the results obtained in biopsied patients.

As expected, the presence of three at-risk variants (3NRV) independently correlated with failed OCR in PBMC when we combined the Discovery and Fibroscan-MASLD cohorts (overall cohort n = 89) (Table S11). *p* < 0.0001, β = −79.05 and 95%CI: −96.29–−61.82). Moreover, in the attempt to define the contribution of the individual mutations, we observed a stronger impact of MBOAT7 and TM6SF2 SNPs on circulating respirometry (Table S11). MBOAT7 T allele, yes: *p* < 0.0001, β = −30.46, and 95%CI: −44.64–−16.28; TM6SF2 T allele, yes: *p* = 0.0002, β = −29.02 and 95%CI: −43.76–−14.28). Finally, to determine whether serum OCR may represent a specific biomarker of MASLD severity (also independently from genetics), we evaluated mitochondrial respiration in the PBMCs of 44 patients affected by unrelated liver diseases encompassing AAT, HH, ALD and autoimmune hepatitis (MASLD-unrelated cohort). Serum OCR was similar between the unrelated liver diseases, and in all these etiologies, OCR was higher compared to that observed in both the biopsied-Discovery and Fibroscan-MASLD cohorts (*** *p* < 0.0001 vs. Biopsied-MASLD and Fibroscan-MASLD) (Figure 8F). According to the multivariable analysis adjusted for age and sex, a lower serum OCR was definitively associated with both biopsied and non-invasively assessed MASLD, thereby highlighting that impaired serum respirometry is specific for MASLD (Table S12. Biopsied-MASLD vs. Unrelated MASLD: *p* = 0.01, β = −19.61 and 95%CI: −34.56–−4.65; Fibroscan-MASLD vs. Unrelated MASLD: *p* = 0.0008, β = −20.29 and 95%CI: −31.82–−8.75; Overall MASLD vs. Unrelated MASLD: *p* = 0.03, β = −16.61 and 95%CI: −31.76–−1.46).

## 4. Discussion

In the present study, we examined the linkage between genetics and mitochondrial failure, both guilty players in MASLD pathogenesis [22,23]. MASLD is currently the most common chronic liver disorder in the entire world, and a liver biopsy still remains the gold standard for the diagnosis of its progressive forms. Challenged by the rising prevalence of MASLD and poor diagnostic and therapeutic strategies, newly non-invasive methodological approaches have been proposed to estimate the severity of liver disease [14]. One of the key steps which features the MASLD switch towards MASH is characterized by mitochondrial dysfunction due to heighten fat accumulation. This results in an unbalanced mitochondrial lifecycle that translates to an aberrant mitochondrial number, morphology, and activity, worsening progressive liver damage. Furthermore, excessive ROS generation driven by mitochondrial failure promotes hepatic fibrosis, triggers lipid peroxidation of PUFAs and produces aldehyde byproducts, thereby exacerbating both cellular damage and death. Thus, oxidative stress creates a vicious cycle by impairing mitochondrial functions and promoting more ROS production, triggering MASLD progression [37]. The disequilibrium of mitobiogenesis and the consequent accumulation of failed mitochondria have been detected in hepatic tissues of both MASH individuals and murine models, thereby underling that MASLD may be considered a mitochondrial disorder. In addition, it has been described that a high release of ccf-mtDNA from damaged liver organelles along with altered serum respirometry correlates with progressive MASLD, thus suggesting the potential use of circulating mitochondrial molecules as noninvasive biomarkers [14,21].

Notoriously, MASLD is a multifactorial disorder characterized by a strong hereditable component [22,38]. Furthermore, we demonstrated that the co-presence of *PNPLA3*, *MBOAT7* and *TM6SF2* at-risk variants in 1380 MASLD subjects hugely predisposes to HCC development, highlighting the usefulness of polygenic risk scores in predicting disease severity. By exploiting a genetic in vitro model, we demonstrated that the deletion of *MBOAT7* and *TM6SF2*, alone and mostly combined, overflowed intracellular lipid accumulation and in parallel triggered mitochondrial derangement, resulting in altered morphology and activity that promoted the advanced hepatic damage [23].

Since MASLD occurs as a mitochondrial disease and the *PNPLA3, MBOAT7 and TM6SF2* loss-of-function mutations seem to achieve a role in organelles’ dysfunction, in this study, we firstly investigated the impact of gene deletion on mitochondrial dynamism and integrity in KO HepG2 cells. Then, we explored whether the restoration of the WT proteins in this in vitro model could rescue mitochondrial activity and morphology and dampen the release of molecules such as ccf-mtDNA. Finally, we compared the hepatic bioenergetic profile with the circulating one in patients carrying the three at-risk variants with the purpose to define a prognostic and specific signature of progressive MASLD in these individuals.

We observed impaired mitochondrial dynamism in HepG2 KO cells which was more evident when both the *MBOAT7* and *TM6SF2* genes were deleted. Then, we performed lentiviral transfection to overexpress WT MBOAT7 and TM6SF2 proteins alone or in combination in HepG2 KO cells. The WT overexpressed models strongly reduced intracellular fat accumulation, similarly to what was reported by Sharpe and colleagues in male C57BL6/J-*MBOAT7^+/+^* mice and by Pant et al. in Huh-7-*TM6SF2 WT^+/+^* cells [39,40]. Interestingly, the WT overexpression of *MBOAT7* or *TM6SF2* in the *MBOAT7^−/−^TM6SF2^−/−^* clone brought out a glass effect, resulting in a more conspicuous lipid reduction in *MBOAT7^+/+^TM6SF2^−/−^* than *MBOAT7^−/−^TM6SF2^+/+^*. These data confirmed the link between the macrosteatotic phenotype and the MBOAT7 loss-of-function mutation previously demonstrated in *MBOAT7^−/−^* cells, which resulted in the shift in phosphatidylinositols toward the synthesis of saturated and monounsaturated triacylglycerols followed by the consequent induction of DNL. Contrariwise, *TM6SF2* KO induced the formation of smaller lipid droplets (LDs) by increasing the synthesis of triacylglycerols enriched in saturated and monounsaturated fatty acid chains [23].

As mentioned above, when combined, *TM6SF2* and *MBOAT7* KO models impair mitochondrial morphology and biomass [23]. Consistently, in the present study, we observed that the deletion of *MBOAT7* or *TM6SF2* alone and mainly together promoted fission while inhibiting both fusion and mitophagy, showing unbalanced mitobiogenesis and an increased assembly of misshapen and failed organelles as we recently demonstrated in the hepatic biopsies of MASLD patients stratified according to their genetic background [41]. Conversely, WT *MBOAT7* and *TM6SF2* co-overexpression rebalanced the mitochondrial life course, thus ensuring a physiological organelle morphology, architecture, and activity. These results corroborate previous findings according to which a high number of mitochondria may correlate with impaired organelles’ dynamics and progressive MASLD [42,43,44,45]. Increased mitochondrial biomass due to the accumulation of globular mitochondria in KO cell lines unlike the physiological *spaghetti-like* shape in WT overexpressed cells did not mirror higher activity of the organelles. In keeping with these data, fibrotic mice fed with a high-trans-fat, high-fructose and high-cholesterol (AMLN) diet showed an increased number of disrupted mitochondria which were featured by low OXPHOS capacity, a loss of the cristae architecture and reduced expression of *Mnf1* and *Opa1* [46]. Furthermore, Zhang et al. demonstrated that *Mnf1* expression was reduced in 34 HCC patients and in MHCC97-H cells and inhibited cell proliferation, migration and invasion, thereby playing a crucial role in impeding HCC development [47].

In another study, MASH patients showed high mitochondrial diameters, intra-mitochondria crystalline inclusions and granules in the matrix, which correlated with both mitochondrial swelling and OXPHOS failure [48]. Previous evidence has reported that in the hepatic tissue of patients and animal models with MASLD, the mitochondrial respiratory chain capacity is reduced. Compared to healthy individuals, patients with MASLD have a reduction in respiratory chain activity of 37% in complex I, 42% in complex II, 30% in complex III, 38% in complex IV, and 58% in complex V [49,50]. Consistently, we observed that *MBOAT7* and *TM6SF2* double KO decreased OXPHOS capacity, ATP production, ketogenesis and β-oxidation. The mitochondrial activity was rescued by the overexpression of the WT proteins and was paralleled by a reduced release of ccf-mtDNA. Moreover, KO cells promoted tumorigenic switching in terms of enhanced oxidative stress, anaerobic glycolysis, proliferation and invasiveness, which were strongly attenuated in WT overexpressed models.

Notably, we found that the *TM6SF2* loss-of-function mutation more than the *MBOAT7* one may be mostly involved in mitochondrial derangement by further increasing the total number of misshapen and failed mitochondria. In corroboration with our results, it has been demonstrated that TM6SF2 deficiency reduced the amount of polyunsaturated fatty acids (PUFAs), along with alterations in mitochondrial β-oxidation in Huh-7 cells, whereas it induced changes in endoplasmic reticulum architecture in the small intestine of zebrafish, supporting that the *TM6SF2* loss-of-function mutation impacts both organelles’ morphology and activity [30,32]. Concerning *PNPLA3*, it has been recently suggested that the I148M variant may be a gain-of-function mutation that promotes hepatic steatosis by accumulating lipid droplets and inhibiting other lipases in an ABHD5-dependent manner [51]. In our in vitro model, we found that the presence of the I148M variant alone did not affect the mitochondrial dynamics. However, evidence from the literature reported that the overexpression of I148M in Huh-7 hepatoma cells was correlated with high levels of lactate and γ-glutamyl amino acids, hallmarks of metabolic switching and mitochondrial dysfunction, respectively [27]. Therefore, in order to investigate the possible crosslink between *PNPLA3* and organelles’ maladaptation, we upregulated the I148M-mutated protein in HepG2 cells in order to force the mutation effect and in Hep3B cells which were WT for the I148M variant (HepG2^I148M+/+^ and Hep3B^I148M+/+^). The in vitro overexpression of I148M enhanced LD accumulation, reduced mitochondrial functions in terms of the OXPHOS rate and resulted in ATP reduction in both cell lines. This caused boosted oxidative damage as well as the release of lactate and ccf-mtDNA, suggesting the involvement of the I148M mutation in mitochondrial failure.

To sum up, dysfunctional metabolism encompassing lipid accumulation, oxidative stress and mitochondrial aberrances alongside genetics lies at the center of MASLD pathogenesis [20]. However, we firstly demonstrated a connection between MASLD-related genetics and mitochondrial dysfunction, and in this paper, the in vitro results demonstrated that genetics impacts mitobiogenesis, thereby giving an explanation of higher organelle content and impaired activity.

High ccf-mtDNA levels in body fluids were correlated with advanced MASLD stages, underling their use as a novel, non-invasive, cheaper and repeatable MASLD diagnostic strategy [14]. We observed in hepatoma cells that the silencing of the *MBOAT7* and *TM6SF2* genes led to an enhanced release of ccf-mtDNA, whose levels were conversely reduced in overexpressed cells. In addition, ccf-mtDNA correlated with impaired respiration in terms of lower activity of complex V (ATP synthase) and reduced OCR.

Therefore, we tried to establish whether the circulating mitochondrial bioenergetic profile accurately reflects the hepatic one to support the potential use of molecules derived from damaged mitochondria (i.e., ccf-mtDNA) as a biomarker of advanced diseases in genetically predisposed individuals. Consistently with our in vitro findings, the liver biopsies of patients carrying 3NRV exhibited a strong reduction in mitochondrial activity, resulting in a low OXPHOS rate. Interestingly, hepatic and circulating mitochondrial respiration was strongly reduced in MASLD patients with MASH-fibrosis supported by enhanced ROS generation, thus emphasizing mitochondrial dysfunction as a potential signature of advanced diseases. Notably, the PBMCs’ bioenergetic profile thoroughly reflected the hepatic one and showed a strong prognostic value for MASH-Fibrosis in 3NRV carriers (n = 44) [21,52,53]. Accordingly, serum respirometry was lower in 3NRV Fibroscan-MASLD patients, exhibiting again a sharp accuracy in predicting fibrosis. This evidence highlighted that serum OCR has a similar capacity to predict fibrosis in both biopsied and non-invasively assessed MASLD patients with a genetic predisposition. Additionally, impaired serum respirometry seems to be specific to MASLD since its levels were higher in unrelated liver disease patients (n = 45) and unchanged across different etiologies.

This study proves that restoring PNPLA3, MBOAT7, and TM6SF2 activities could counteract MASLD, pointing to them as potential therapeutic targets at the RNA level for personalized therapy. The targeting of the PNPLA3 I148M variant in a mouse model using tri-antennary N-acetyl galactosamine (GalNAC3) conjugated with an antisense RNA oligonucleotide (ASO) decreased hepatic inflammation, steatosis and fibrosis [54]. In another recent study, targeting Pnpla3 in 148M knock-in mice using Adeno-associated viral AAV-mediated shRNA reduced hepatic triglyceride content [55].

Focusing on our results, suitable approaches could be represented by gene therapy strategies based on the gene transfer that can introduce a WT copy to recover the function of the protein. For instance, the AAV-mediated delivery of a permanently active mutant form of human carnitine palmitoyl transferase 1A (hCPT1AM) in the liver of mice fed a high-fat diet enhanced liver fatty acid oxidation [56]. Furthermore, the AAV8-mediated gene transfer approach that allows for long-term hepatic SIRT1 overexpression counteracted high-carbohydrate diet-induced MASLD and improved whole-body metabolism in adult mice [57]. Supported by these literature results, we may delineate a new proof of concept regarding the applicability of gene transfer therapy in genetics-based MASLD management to prevent its progressive forms.

## 5. Conclusions

To conclude, the novel aspects of this study are twofold. We confirmed the contribution of *PNPLA3*, *MBOAT7*, and *TM6SF2* loss-of-function variants to mitochondrial dysfunction that features progressive MASLD, suggesting that their restoration which reverses the failure phenotype may be a successful therapeutic strategy.

Secondly, we demonstrated for the first time that mitochondrial damage in PBMCs is the same as in the liver in MASLD patients, and it is specific for the disease, thus suggesting in a pioneering fashion the use of circulating mitochondrial biomarkers (i.e., mt-ccf and a bioenergetic index) to foresee disease severity in genetically predisposed individuals.

## Data Availability

Data is contained within the article or Supplementary Materials.

## Supplementary Materials

The following supporting information can be downloaded at https://www.mdpi.com/article/10.3390/antiox14060618/s1, Figure S1: MBOAT7 and/or TM6SF2 WT overexpression improves lipid overload due to their silencing; Figure S2: MBOAT7 and/or TM6SF2 WT overexpression rebalances the mitochondrial lifecycle and turnover; Figure S3: MBOAT7 and/or TM6SF2 WT overexpression in knock-out models restore the mitochondrial functions; Figure S4: MBOAT7 and/or TM6SF2 WT restore in knock-out cells attenuates the tumorigenic phenotype; Table S1: Clinical features of the MASLD biopsied patients (Discovery cohort); Table S2: Clinical features of patients with non-invasive diagnosis of MASLD (Fibroscan-MASLD cohort); Table S3: Clinical features of the patients belonging to the Unrelated Liver Disease Cohort; Table S4: (A) List of TaqMan Probes and (B) sequence of primers used in Quantitative Real-Time PCR Experiments; Table S5: List of Antibodies and Relative Dilutions Used in Western Blot (WB) and Immunocytochemistry (ICC) Experiments; Table S6: Association between NRV = 3 and hepatic oxidative stress, citrate synthase activity, mitochondrial complexes activity in the Discovery cohort; Table S7: Association between NRV = 3 and PBMCs oxidative stress, citrate synthase activity, mitochondrial complexes activity in the Discovery cohort; Table S8: Association between 3NRV and reduced respiration (complex I/IV) both in liver and PBMCs in the Discovery cohort; Table S9: Association between 3NRV and reduced respiration (complex II/IV) both in liver and PBMCs in the Discovery cohort; Table S10: Association between 3NRV and reduced PBMCs respiration (complex I/IV) in the Fibroscan-MASLD cohort; Table S11: Association between 3NRV and reduced PBMCs respiration (complex I/IV) in the overall MASLD cohort; Table S12: Specific association between reduced serum OCR and MASLD.

## Abbreviations

The following abbreviations are used in this manuscript: Metabolic dysfunction-associated steatotic liver disease (MASLD); metabolic dysfunction-associated steatohepatitis (MASH); patatin-like phospholipase domain-containing 3 (PNPLA3); transmembrane 6 superfamily member 2 (TM6SF2); membrane bound o-acyltransferase domain-containing 7 (MBOAT7); peripheral blood mononuclear cells (PBMCs); mitochondria (mt); adenosine triphosphate (ATP); mitochondrial damage-associated molecular patterns (Mito-DAMPs); mitochondrial DNA (mtDNA) fragments (ccf-mtDNA); patatin-like phospholipase domain-containing 3 (PNPLA3); transmembrane 6 superfamily member 2 (TM6SF2); membrane-bound o-acyltransferase domain-containing 7 (MBOAT7).
